# Mortality trends and geographic distribution of kidney cancer in Peru: a secondary analysis

**DOI:** 10.1186/s12894-023-01208-7

**Published:** 2023-03-29

**Authors:** J. Smith Torres-Roman, Gabriel De la Cruz-Ku, Valeria Juárez-Leon, Delahnie Calderón-Solano, Janina Bazalar-Palacios, Carlo La Vecchia, Paulo S. Pinheiro

**Affiliations:** 1 Escuela Profesional de Medicina Humana, Universidad Privada San Juan Bautista, Filial Chincha, Ica, Peru; 2 Universidad Científica del Sur, Lima, Perú; 3 Latin American Network for Cancer Research (LAN–CANCER), Lima, Peru; 4 Universidad Privada Norbert Wiener, Lima, Peru; 5 Department of Clinical Sciences and Community Health, Università degli Studi di Milano, Milan, Italy; 6 Sylvester Comprehensive Cancer Center, University of Miami School of Medicine, Miami, USA; 7 Department of Public Health Sciences, University of Miami Miller School of Medicine, Miami, USA

**Keywords:** Kidney cancer, Peru, Regions, Mortality

## Abstract

**Background:**

The incidence of kidney cancer has been increasing worldwide, with variable patterns in mortality due to improved diagnostic techniques and increased survival. The mortality rates, geographical distribution and trends of kidney cancer in South America remain poorly explored. This study aims to illustrate mortality by kidney cancer in Peru.

**Methods:**

A secondary data analysis of the Deceased Registry of the Peruvian Ministry of Health database, from 2008 to 2019 was conducted. Data for kidney cancer deaths were collected from health facilities distributed throughout the country. We estimated age-standardized mortality rates (ASMR) per 100,000 persons and provided an overview of trends from 2008 to 2019. A cluster map shows the relationships among 3 regions.

**Results:**

A total of 4221 deaths by kidney cancer were reported in Peru between 2008 and 2019. ASMR for Peruvian men ranged from 1.15 to 2008 to 1.87 in 2019, and from 0.68 to 2008 to 0.82 in 2019 in women. The mortality rates by kidney cancer rose in most regions, although they were not significant. Callao and Lambayeque provinces reported the highest mortality rates. The rainforest provinces had a positive spatial autocorrelation and significant clustering (p < 0.05) with the lowest rates in Loreto and Ucayali.

**Conclusion:**

Mortality by kidney cancer has increased in Peru, being a trend that disproportionally affects more men than women. While the coast, especially Callao and Lambayeque, present the highest kidney cancer mortality rates, the rainforest has the lowest rates, especially among women. Lack of diagnosis and reporting systems may confound these results.

## Background

Kidney cancer is the 13th most common cancer worldwide, with more than 430,000 new cases (2.2% of all new cancers) and 179,000 deaths (1.8% of all cancer mortality) estimated in 2020 [1]. Renal cell carcinoma is the most frequent histological type of kidney cancer and is more frequent in men, in overweight and obese individuals, and in persons with a history of smoking, hypertension, and chronic kidney disease [2].

Recent reports have raised concern of the increasing rates of kidney cancer and its associated mortality rates in Latin America [3]. In 2017, Bai et al. [4] reported that the mortality rate of kidney cancer in Latin America was high with 4.28 deaths per 100,000. Similarly, other reports in the region have demonstrated that the incidence of kidney cancer has been increasing over the last 10 years, creating the need for greater understanding of this phenomenon [4].

Moreover, some reports from the Lima Metropolitan Cancer Registry [5–7], in Peru, have described an increase in the incidence and mortality rates for this disease. For example, from 2004 to 2005 to 2013–2015, the mortality rates for men ranged from 2.4 to 3.9, whereas in women they ranged between 1.0 and 1.5, respectively [7–9]. The incidence of urological cancers and chronic kidney disease has been identified as high in Peru, but little is known about the epidemiological distribution of kidney cancer in this country [10, 11]. Moreover, in some provinces, such as Huancavelica, mercury levels are high, being an important factor for the development of renal diseases [12] and the need for further studies. Therefore, we sought to examine the geographic distribution of mortality by kidney cancer and identify gender-related differences in Peru.

## Methods

### Design and study setting

A secondary analysis was conducted using the National Informatic System of Deaths (SINADEF in Spanish). SINADEF is a collaboration of the Ministry of Health, the National Institute of Statistics and Informatics and the National Registry of Identification and Civil Status, which registers the data of deceased persons, the origin of the death certificate and the statistical report [13]. Cases of kidney cancer-related deaths reported between 2008 and 2019 were included in the study. The diagnosis of kidney cancer was identified by code C64 according to the International Classification of Diseases (ICD), 10th Revision [14]. The demographic data were collected by the Network of the Ministry of Health comprised by health care facilities distributed in the 24 provinces of Peru. The information is available through its online platform: http://www.minsa.gob.pe/portada/transparencia/solicitud/.

Peru is located in the Andean region of South America, South of the equator and on the coast of the Pacific Ocean. It is divided into 24 provinces, grouped into 3 geographical regions: coast, highlands, and rainforest and has 31 million inhabitants unequally distributed among its 3 regions. While the coast only covers 12% of the national territory, it is the most populated region with approximately 56% of the total (around 17 million inhabitants). In contrast, the highlands cover approximately 28% of the national territory and include 30% of the total population (around 9 million inhabitants). The rainforest (Peruvian Amazon) is the largest region of the country; accounting for 60% of the national territory but only contains 14% of the total population (around 5 million inhabitants) [15].

Peru is a low/middle income country, with a life expectancy at birth of 72.5 years for men and 77.7 years for women and an infant mortality rate of 17 per 1,000 live births, which has significantly improved over the last two decades. Over the same period, Peru has experienced strong economic growth; accompanied by marked migration to urban centers (mainly on the coast), a reduction of the population living in extreme poverty and a shift in mortality by infectious to non-communicable diseases. Along this period of transition, overweight and obesity has been increasing [16]. However, advances in health care delivery systems and dissemination of health access have been slow, unequally affecting people living in rural areas including the highlands and rainforest [17].

### Ethical considerations

This manuscript is based on administrative databases and does not use any personal identifiable information.

### Statistical analysis

Age-standardized mortality rates (ASMR) were estimated per 100,000 person-years using the direct method and the world SEGI standard population, as indicated by the World Health Organization [18]. For the denominator, we used the population in five-year age groups, provided by the National Statistics Institute [19]. We analyzed the mortality rates for kidney cancer by sex in the last five-years per each province, with the objective of reporting the provinces with the highest mortality rates in recent years. Trends in mortality were analyzed using the Joinpoint regression Program Version 4.7.0 [20], to identify the occurrence of possible Joinpoints, i.e., significant changes in slopes. The final model selected was the Annual Percentage Change based on the trend of each segment, estimating whether these values were significant (p < 0.05). The significance levels utilized herein are based on the Monte Carlo permutation method [21] and on the calculation of the average propensity to consume of the ratio, using the logarithm of the ratio.

The spatial analysis was conducted with GeoDA software [22]. The spatial analysis was performed using Moran’s I statistic. The map results in a spatial typology consisting of five categories of health regions: (i) ‘high–high’ (positive autocorrelation), (ii) ‘low–high’ (negative autocorrelation), (iii) ‘low–low’ (positive autocorrelation), (iv) ‘high–low’ (negative spatial autocorrelation), and (v) ‘not significant’ indicating that there was no spatial autocorrelation. The value of the Moran index varies between − 1 and + 1, where negative values indicate a spatial conglomerate of territorial units with different values of analysis and positive values indicate a spatial conglomerate of territorial units with similar values of analysis. We used a reference distribution using 999 random permutations to indicate statistical significance.

## Results

**Fig. 1:**
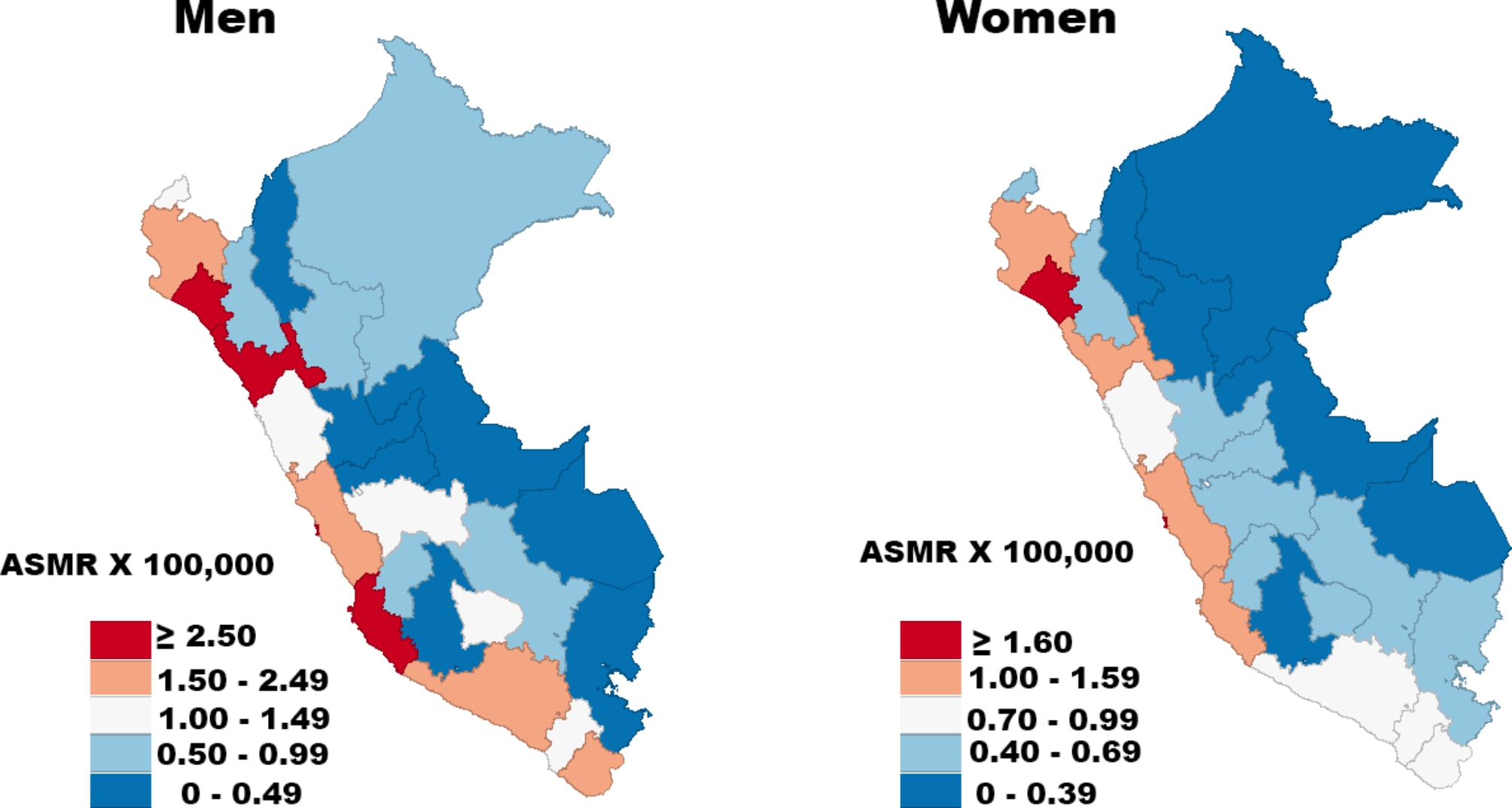
Provincial age-standardized mortality rates per 100,000 person-years for kidney cancer by sex, 2015–2019

A total of 4221 kidney cancer deaths (2706 men and 1515 women) were registered in Peru during the study period. Figure 1 shows the provincial ASMRs per 100,000 person-years for kidney cancer by gender in the last 5 years. The coastal provinces had the highest mortality rates for men, mainly in Lambayeque, La Libertad, Callao e Ica (≥ 2.5 deaths per 100,000), whereas the rainforest provinces had the lowest mortality rates (0 to 0.99 deaths per 100,000). In relation to women, Lambayeque and Callao reported the highest mortality rates for kidney cancer (≥ 1.6 per 100,000), whereas the rainforest and highlands provinces showed the lowest rates (0 to 0.99 deaths per 100,000).

Table 1 reports the joinpoint analysis for kidney cancer in Peru and its regions. Mortality by kidney cancer increased, albeit not significantly, in the coastal and highlands regions of Peru during the study period. However, mortality in the rainforest region could not be evaluated because some years showed zero deaths.

**Table 1:** Joinpoint analysis for kidney cancer from Peru and its regions between 2008 and 2019

Geographical area	Men		Women			
	Years	APC	Years	APC	Years	APC
**Peru**	2008–2019	2.2(− 0.6,5.1)	2008–2019	1.8(0, 3.7)		
**Coast**	2008–2019	2.0(− 1.0,5.1)	2008–2019	1.8(− 0.5,4.1)		
**Highlands**	2008–2019	2.7(− 1.8,7.4)	2008–2019	2.4(− 2.6,7.7)		
**Rainforest**	2008–2019	NA	2008–2019	NA		

NA: Not applicable

**Fig. 2:**
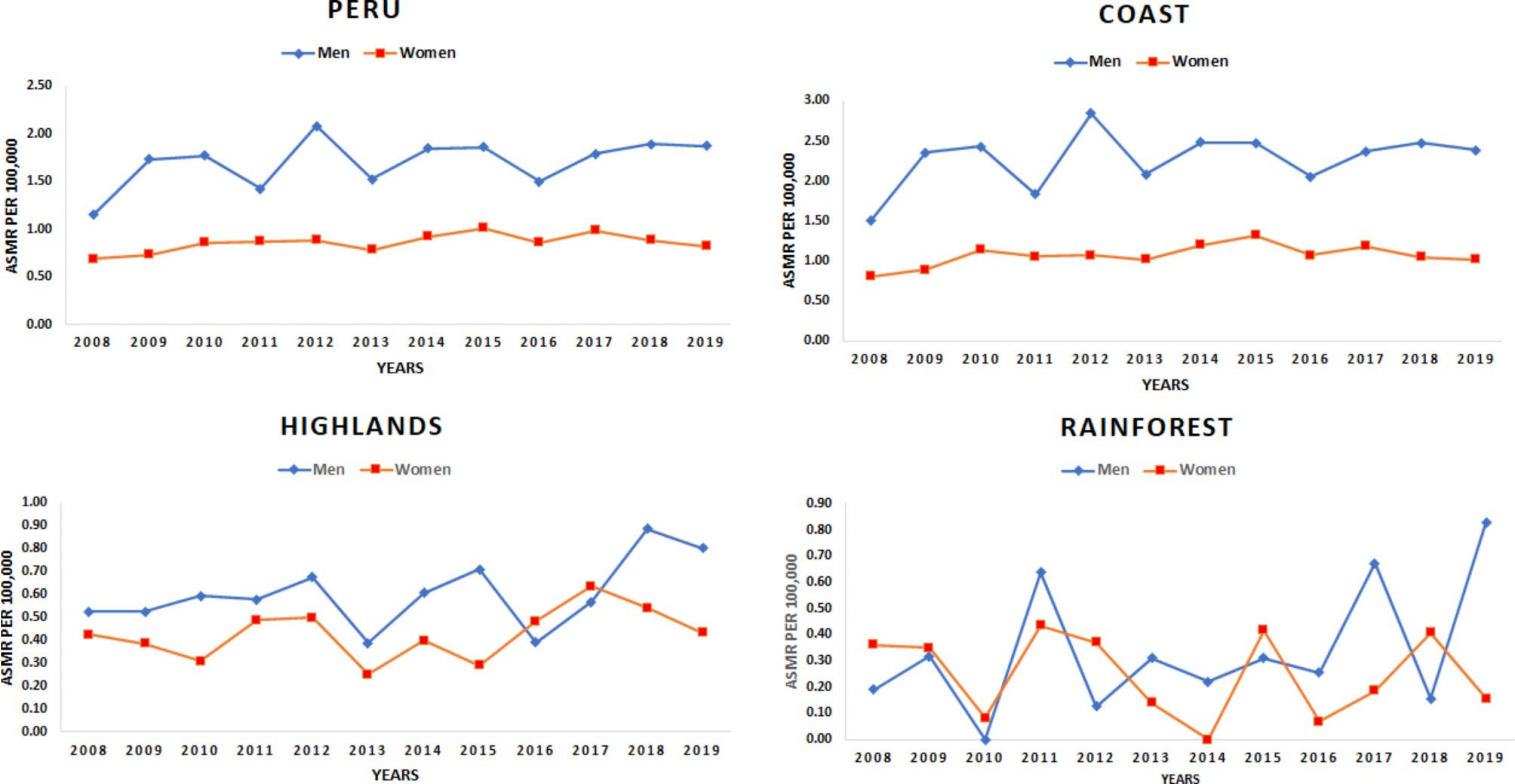
National and regional age-standardized mortality rates per 100,000 person-years for kidney cancer per year and sex, 2008–2019

Figure 2 shows national and regional ASMRs per 100,000 person-years for kidney cancer per year and sex. The men in the coast and highlands regions had the highest mortality rates compared to women. Mortality rates for Peruvian men ranged from 1.15 to 2008 to 1.87 in 2019, and ranged from 0.68 to 2008 to 0.82 in 2019 in women. The rates in men residing in the coastal region ranged from 1.5 to 2008 to 2.39 in 2019, with the highest rate in 2012 (2.84 per 100,000). The rates in men living in the highlands ranged from 0.53 to 2008 to 0.80 in 2019, with 0.43 deaths per 100,000 in women in both 2008 and 2019.

The ASMR (per 100,000 person-years) was estimated in three sub-periods: 2008–2011, 2012–2015, and 2016–2019. Overall, mortality rates in men increased from 1.52 (2008–2011) to 1.76 (2016–2019), representing an increase of 16.1%. Among women, mortality rates increased from 0.79 (2008–2011) to 0.89 (2013–2016), being an increase of 12.7% (Table 2). According to regions, the coast reported the highest mortality rates in all sub-periods among men (2.03 to 2.47 per 100,000) and women (0.97 to 1.15 per 100,000). On the coast, the increases in mortality rates were similar in both men and women. In the highlands, an increase of 19% was reported among men, whereas in women the increase was 30.1%. The lowest rates were reported in the rainforest region; however, men showed the greatest increase (66.9%) between the first (2008–2011) and last subperiod (2016–2019) in this region.

**Table 2:** Age-standardized mortality rates per 100,000 person-year for kidney cancer in men and women in 2008–2011, 2012–2015 and 2016–2019, and the corresponding percentage change

	Men				Women			
	2008–2011	2012–2015	2016–2019	%change(2016-19/2008-12)	2008–2011	2012–2015	2016–2019	%change(2016-19/2008-12)
Peru	1.52	1.83	1.76	16.1	0.79	0.90	0.89	12.7
Coast	2.03	2.47	2.32	14.4	0.97	1.15	1.08	11.1
Highlands	0.56	0.59	0.66	19.0	0.40	0.36	0.52	30.1
Rainforest	0.29	0.24	0.48	66.9	0.31	0.23	0.20	-33.5
Amazonas	0.11	0.42	0.00	-100.0	0.35	0.58	0.00	-100.0
Ancash	0.35	0.81	1.72	395.0	0.47	0.62	0.74	58.0
Apurimac	0.61	0.73	1.10	80.8	0.10	0.24	0.48	360.6
Arequipa	2.12	1.38	2.31	9.0	0.81	0.84	1.03	27.6
Ayacucho	0.26	0.11	0.57	116.5	0.70	0.37	0.34	-51.2
Cajamarca	0.28	0.48	0.37	30.7	0.39	0.20	0.62	57.2
Callao	2.41	4.52	3.52	46.3	1.50	1.66	1.87	24.1
Cusco	0.49	0.23	0.69	41.3	0.14	0.16	0.53	268.7
Huancavelica	0.41	1.28	0.62	52.0	0.16	0.39	0.70	342.7
Huanuco	0.62	0.54	0.37	-40.7	0.56	0.14	0.50	-10.5
Ica	1.92	1.67	2.97	55.0	0.83	0.94	1.25	50.4
Junin	1.02	1.00	1.34	31.0	0.75	0.79	0.52	-30.4
La Libertad	2.01	2.67	2.38	18.4	1.07	1.44	1.27	19.1
Lambayeque	2.16	2.60	2.87	32.5	0.91	1.96	1.73	89.5
Lima	2.19	2.67	2.23	2.0	1.02	1.06	0.96	-5.3
Loreto	0.70	0.37	0.73	3.4	0.32	0.15	0.10	-68.5
Madre de Dios	0.00	0.00	0.24	NA	0.42	0.00	0.34	-18.4
Moquegua	1.81	1.79	1.14	-37.1	0.90	0.25	0.89	-1.4
Pasco	0.58	1.13	0.49	-15.8	0.00	0.36	0.58	NA
Piura	2.03	2.35	2.03	0.2	0.96	1.51	0.93	-3.5
Puno	0.55	0.63	0.36	-34.4	0.36	0.48	0.47	31.3
San Martin	0.00	0.18	0.60	NA	0.14	0.24	0.28	108.3
Tacna	1.78	2.87	2.22	24.3	0.55	0.38	0.97	76.3
Tumbes	2.42	0.67	1.13	-53.4	1.09	0.33	0.26	-76.4
Ucayali	0.19	0.00	0.25	29.8	0.51	0.09	0.39	-23.4

NA: Not applicable

**Fig. 3:**
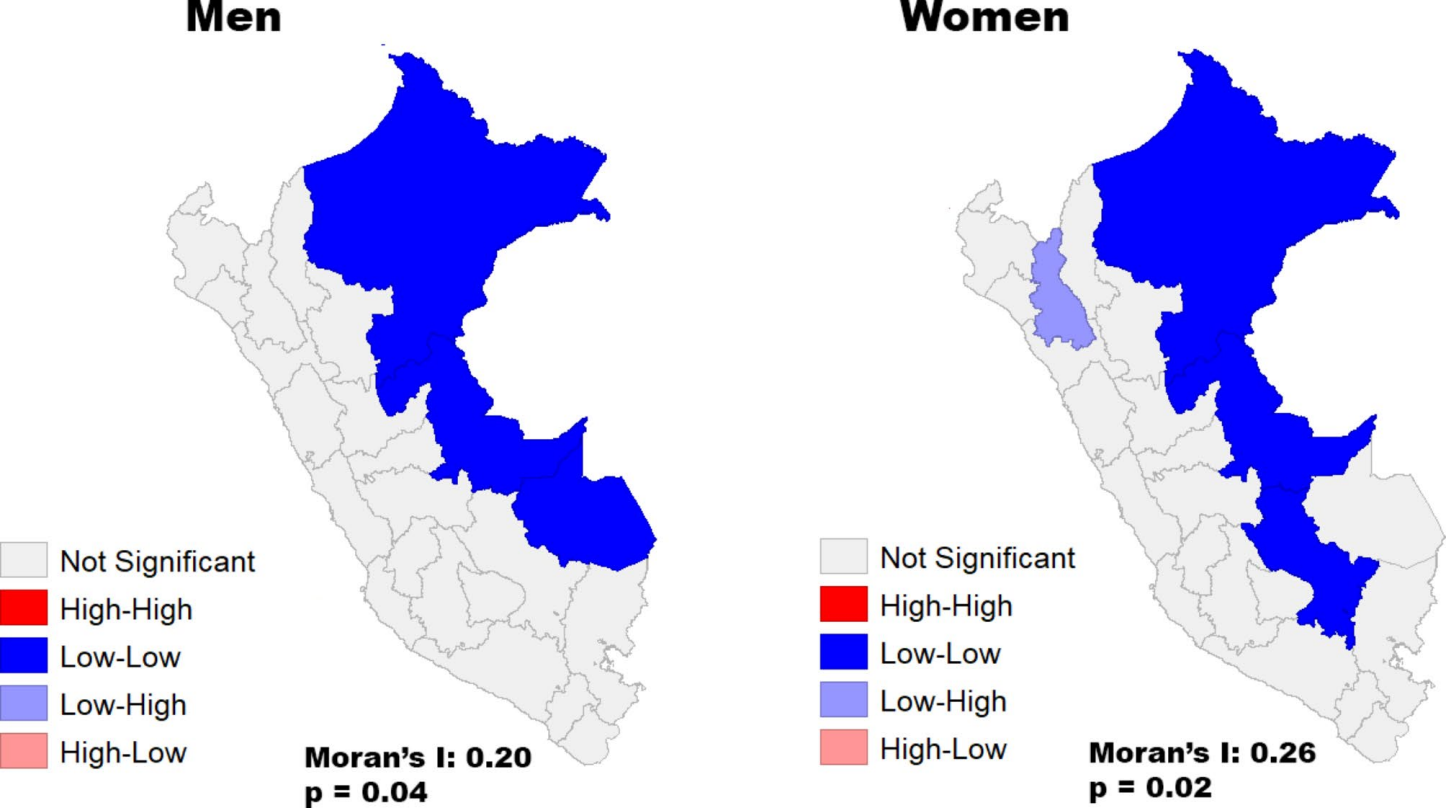
Spatial cluster map of kidney cancer mortality rates by sex for the period 2008–2019

Figure 3 shows a spatial cluster map of kidney cancer mortality rates. From 2008 to 2019 men showed a positive spatial autocorrelation and significant clustering (Moran’s I: 0.20, P = 0.04) with the lowest rates being in the Peruvian North-East (Loreto, Ucayali, and Madre de Dios). For women, a positive autocorrelation was also reported (Moran’s I: 0.26, P = 0.02) with the lowest rates in Loreto, Ucayali and Cusco.

## Discussion

This study is based on an analysis of the kidney cancer death registry in Peru. Using estimations based on ASMR, we found that kidney cancer mortality rates have been increasing in Peru, with variations according to sex and geographical area. While in women, the ASMR increased in the coast and highlands, it decreased in the rainforest. In contrast, the ASMR increased in men, being more notable in the rainforest region. Furthermore, the coast presented the highest ASMR in both sexes, whereas the rainforest region had the lowest mortality rates in both sexes.

Our study identified and quantified an increase in the ASMRs for kidney cancer in both sexes. It is therefore important to consider early action for this disease. In Peru there are around 2 deaths per 100,000 men and around 1 death per 100,000 women. In 2020, GLOBOCAN reported mortality rates close to those reported in our study. For example, the reported rates for men were greater than 2 deaths per 100,000, being greater than 1 death per 100,000 for women [11]. Although these rates are close to those reported by Puerto Rico, Cuba, Panama, Colombia, and Nicaragua, they are low compared to Chile, Argentina, and Uruguay (rates from 4.9 to 7.2 per 100,000 for men and from 1.9 to 2.3 per 100,000 for women) [11]. It is therefore important to control the risk factors for the development of kidney cancer, such as smoking, obesity, hypertension, and chronic kidney disease [23, 24]. In fact, the region with the highest mortality rate is the coast, which has the highest prevalence of the aforementioned risk factors [10, 25, 26]. In high income countries the diagnosis, management and treatment of kidney cancer have substantially improved over the last few decades [27], although with appreciable socio-economic differentials [28]. Nonetheless, Peruvian mortality rates remain lower than those in most high-income countries, likely indicating under registration of deaths. Moreover, Peru does not have incidence registries for cancer or studies of survival in this cancer, making it difficult to demonstrate the real status of this neoplasm.

Our study also reports a clear difference in kidney cancer mortality by sex in Peru and its regions.

The coast region had the highest mortality rates among men and women across all subperiods. This can be explained by the fact that cities in Peru’s coastal region have higher levels of economic development than other cities. With greater development comes a higher prevalence of risk factors such as smoking, having a high BMI, being inactive, and having hypertension, which may be more prevalent in developed areas [29]. Furthermore, increases in kidney cancer incidence may be due in part to a lack of health staff, limited access to health services, and limitations in diagnostic and treatment options such as diagnostic imaging. On the other hand, the increase in mortality was not as severe in the Sierra and Selva regions, where it is known that the harmful effects of illegal mining, the main occupation of its Inhabitants, and its short- and long-term consequences are a concern [30]. However, when compared to the coastal region, this region has a high certification rate or data loss. As a result, civil registration and vital statistics systems are an important source of information and evidence for monitoring population health, identifying health priorities, and planning interventions to reduce disease mortality.

Finally, as we see an increase in the incidence and mortality of kidney cancer, we need to focus on developing strategies for early detection. Additional campaigns and medical care are needed to reduce the prevalence of smoking, obesity, and hypertension, all of which are major risk factors for kidney cancer. It would improve the situation further without impeding equal treatment access, particularly through new targeted therapies.

The present study has several limitations, such as a loss of data or underreporting of deaths in some departments. In addition, inaccurate knowledge of the number of deaths in each department makes it impossible to determine the real incidence of renal cancer. Moreover, no study has analyzed the overall survival of this disease, thereby making it impossible to show the reality of this disease in Peru. Some departments such as Pasco, San Martin, or Madre de Dios reported zero deaths in some of the years studied, perhaps due to loss of data in these departments, and thus, we could not identify the percentage change that occurred in these geographic areas. The main interest of our study is that this is the first study to report mortality by renal cancer in Peru, in addition to studying deaths by regions and departments by sex. It also allowed identification of geographic areas in which epidemiological surveillance of risk factors for this disease is needed.

## Conclusion

Our study describes trends in kidney cancer mortality in Peru and its geographical areas. Mortality by kidney cancer has increased in Peru, mainly in men compared to women. The highest mortality rates by kidney cancer were observed in the coastal region, whereas the rainforest had the lowest mortality rates.

## Data Availability

The datasets used during the current study are available upon request from: https://www.minsa.gob.pe/portada/transparencia/solicitud/ or also be requested from the corresponding author.

## Abbreviations

SINADEF: National Informatic System of Deaths
ICD: International Classification of Diseases
ASMR: Age-standardized mortality rates
